# Symptomatic progression-free survival as an emerging patient-centered endpoint in multiple myeloma: a secondary analysis of MagnetsiMM-3 trial data

**DOI:** 10.1186/s12885-025-14724-6

**Published:** 2025-08-08

**Authors:** Martin Kortüm, Sebastian Theurich, James Farrell, Graham Jackson, Gordon Cook, Joseph C. Cappelleri, Olivia Ashman, Shanti Neff-Baro, Aline Gauthier, Khalil Jewiti-Rigondza, Alaeddine Sidhom, Ashraf Chaudhary, Christof Scheid

**Affiliations:** 1 Würzburg University Hospital, Oberdürrbacherstr.6, 97080 Würzburg, Germany; 2 Department of Medicine III, LMU University Hospital, Munich, Germany; 3 Pfizer Ltd, Dublin 4, Ireland; 4 Freeman Hospital, Newcastle upon Tyne, UK; 5 Cancer Research UK Clinical Trials Unit, Leeds Institute of Clinical Trials Research, University of Leeds Hospital, Leeds, UK; 6 Pfizer Inc, New York, USA; 7 Pfizer Ltd, Tadworth, UK; 8 Amaris Ltd, Lyon, France; 9 Amaris Ltd, Barcelona, Spain; 10 Cologne University Hospital, Cologne, Germany

**Keywords:** Composite endpoint, Symptomatic progression-free survival, Patient-reported outcomes, Progression, Systematic literature review

## Abstract

**Background:**

There is a need to better reflect the patient experience in multiple myeloma (MM), beyond just clinical progression, with the use of a novel endpoint that combines disease progression with patient-reported outcomes.

**Methods:**

A systematic literature review (SLR) identified relevant MM symptoms, which were narrowed down by experts using a Delphi panel. A post-hoc analysis of the MagnetisMM-3 trial of elranatamab assessed the impact of change in patient-reported outcomes (PROs) on the risk of disease progression or death. A composite endpoint for symptomatic progression-free survival (SPFS) combined these symptoms and PFS (PFS event plus 10-point worsening in pain, fatigue, or poor mobility within 28 days).

**Results:**

The SLR identified 16 symptoms from 34 publications. Delphi consensus was that drowsiness, fatigue, pain, and poor mobility are linked to disease progression. Three symptoms had a significant association with PFS: A 10-point worsening on the 100-point scales for pain, fatigue, and poor mobility corresponded to a 22%, 20%, and 31% increase in risk of progression, respectively. Median SPFS was not reached (95% CI 12.2- not reached).

**Conclusions:**

SPFS is the first composite endpoint in MM that combines PFS and clinically relevant PROs. The patient-centric approach in the conception of this endpoint allows for a nuanced and comprehensive understanding of the MM patient experience.

**Supplementary Information:**

The online version contains supplementary material available at 10.1186/s12885-025-14724-6.

## Introduction

Multiple myeloma (MM) is a relapsing-remitting blood cancer, which is characterized by an aberrant growth of the plasma cells in the bone marrow. It often affects other organs, leading to bone damage, renal insufficiency and hypercalcemia, resulting in symptoms such as fatigue, bone pain, and anemia [1, 2]. 

MM is a globally prevalent hematological cancer, representing 1% of all cancer diagnoses, making it the second most common blood cancer with a median overall survival (OS) of 6 years after diagnosis [3–5]. The age-standardized global incidence of MM is 2.35 per 100,000 persons per year with a mortality rate of 1.51 per 100,00 persons globally in 2022 [6]. 

Currently, OSis the most used and meaningful endpoint in MM clinical trial designs; however, challenges remain in obtaining mature OS data. As a result, progression-free survival (PFS) and minimal residual disease (MRD) have been used as endpoints in clinical trials, with some studies showing a moderate to strong correlation with OS [7]. 

However, MM has also been linked to symptom burden, with over half of patients expressing concerns related to eating and nutrition, exercise and physical activity, general physical movement (e.g., walking, climbing stairs, lifting), fatigue, and pain or physical discomfort [1]. As these patient-reported outcomes (PROs), by definition, come directly from the patient experience, the question arises whether clinical outcomes alone sufficiently encapsulate the patient experience. Previously, studies have examined the relationship between PROs and clinical disease progression in other oncological areas, such as metastatic colorectal, advanced gastrointestinal, lung, and genitourinary cancers; however, study of this association in MM is limited [9–11]. Notable exceptions include a study by Knop et al., which investigated joint models of PFS and PROs among patients with relapsed/refractory multiple myeloma (RRMM) [12]. Data from six clinical trials revealed that worsening pain and fatigue were consistently associated with an increased risk of disease progression or death.

To our knowledge, while the association between PROs and PFS has been previously explored, no prior studies have developed a composite endpoint integrating both measures in MM. This highlights the need for a novel endpoint going beyond clinical progression that captures the full patient experience in evaluating MM treatments.

This study aimed to define and develop a novel composite efficacy endpoint, symptomatic PFS (SPFS), for patients with MM, incorporating both patient-reported symptoms and clinical disease progression and survival. The process included a systematic literature review (SLR) and Delphi panel to identify relevant symptoms, followed by a post-hoc analysis of patient-level data from a phase II clinical trial using advanced joint modelling techniques to construct and evaluate the SPFS endpoint. Ultimately, the goal was to demonstrate the value of this patient centric measure in reflecting the patient experience among patients with MM who experience PFS to aid health-technology assessment (HTA) and payer decision-making.

## Materials and methods

The primary objective of this study was to develop a patient-centric composite endpoint that integrates disease progression and patient-reported symptoms in patients with MM. The study was conducted in three phases: first, an SLR was conducted to identify relevant MM symptoms, which were subsequently refined by a panel of MM experts using the Delphi methodology [13]. Second, a joint model was developed utilizing data from the single arm MagnetisMM-3 trial of elranatamab (NCT04649359) to evaluate the impact of change from baseline in PROs on the risk of disease progression or death [14–16]. Finally, a composite endpoint for SPFS was established by combining the identified symptoms withPFS, providing a comprehensive assessment of both disease burden and patient experience.

### Identification of relevant MM symptoms

An SLR was conducted with a cutoff date of April 26, 2023 to identify symptoms important to patients and potentially linked to the risk of MM progression. In addition, PRO instruments assessing each relevant symptom were reviewed. The SLR included studies involving adult patients (≥ 18 years) with MM, both treatment-naive and previously treated, and was conducted in accordance with the Cochrane and Centre for Reviews and Dissemination guidance documents for undertaking systematic reviews to ensure publication-acceptable standards [17]. The research questions were formalized according to the Population, Intervention, Comparator, Outcomes and Study design (PICOS) framework, displayed in Table 1, with the electronic database search strategy detailed in Additional file 1.

**Table 1:** SLR eligibility criteria

Category	Inclusion criteria	Exclusion criteria
Population	Adult patients (≥ 18 years) with MM (including treatment naïve as well as those treated with any line of treatment)	Pediatric patients Patients with smoldering MM, AL-amyloidosis, Waldenstrom macroglobulinemia and Plasma cell leukemia Animal/in vitro
Intervention/ Comparator	Any pharmacological therapy	Non-pharmacological therapy
Outcomes	Symptoms/events depicting progression, and those considered most important to patients, including *but not restricted to*: Fatigue Bone pain Mobility problems Cognitive symptoms Reduced energy Other MM symptoms Note: Any validated PROs measuring symptoms or events were shortlisted.	Studies reporting no outcomes of interest
Study design/publication type	Any studies reporting disease-specific symptoms and treatment-related side effects (including RCTs, observational studies, qualitative and patient preference studies) Guidelines and disease-specific consensus documents Related systematic and targeted literature reviews	Non-peer-reviewed articles Case reports/case studies/case series Editorials/commentary/notes/letters/news articles Conference abstracts with limited information
Language	English language	Language other than English
Geographic scope	No restrictions	Not applicable
Publication year	Full publications: 2013 to April 26, 2023 Conference abstracts: 2020 to April 26, 2023	Publications prior to 2013 Conference abstracts prior to 2020

*MM* multiple myeloma, *PRO* patient-reported outcome, *RCT* randomized controlled trial

Literature was searched electronically in a structured fashion using EMBASE, MEDLINE, MEDLINE In-Process, and the Cochrane library, as well as hand searches for recent conference proceedings, clinical trial registries, and HTA websites. Study selection was performed according to recommendations by 2022 NICE manuals for health technology evaluations and guideline development [18] and G-BA guidance [19]. Relevant data from the included studies was extracted into a pre-specified tabular summary and quality checked by an independent reviewer.

The relevance of symptoms identified in the SLR and their relationship to MM progression was next tested by selected MM experts using a Delphi panel. Delphi is a validated technique that uses multiple iterations of questionnaires designed to develop a consensus [20]. In this study, three rounds of electronic surveys were undertaken with 10 experts in MM (eight clinicians specializing in hematology/oncology, a pharmacist from a hematology/oncology pharmacy, and one payer) [13] where all responses were anonymous, leading to a final consensus. The panel members represented a multinational perspective, from the United States, Germany, the United Kingdom, and Spain. For questions asking panellists whether they agreed or disagreed with a particular statement, the threshold for consensus was preset at ≥ 70% agreement [21]. For questions with categorical scales (i.e., level of importance being described as none (1), low (2), moderate (3), high (4), very high (5)), consensus required all answers to fall within a range of ≤ 1.

### Post-hoc analysis of MagnetisMM-3 (MM-3) data

MM-3 (NCT04649359) is an open-label, multicentre, non-randomized phase II study to evaluate the efficacy and safety of elranatamab in patients with RRMM who are refractory to at least one proteasome inhibitor (PI), one IMiD, and one anti-CD38 monoclonal antibody (mAB) [22]. A post-hoc analysis of data from MM-3 was conducted to test the hypothesis that MM symptoms as measured by PRO scores have an impact on the risk of clinical progression or death as defined by the International Myeloma Working Group (IMWG) criteria and to ultimately construct an SPFS composite endpoint and evaluate this endpoint among MM-3 patients [23]. The MM-3 trial data cutoff for this analysis was March 26, 2024 and database snapshot date May 31, 2024, which represented a median follow-up of approximately 28 months for the overall study population.

### Patient population

The trial included two independent cohorts: the pivotal Cohort A, consisting of participants naïve to B-cell maturation antigen (BCMA)-directed therapies, and Cohort B, comprising those previously exposed to BCMA-directed therapy. The analysis was conducted on the pivotal Cohort A safety population dataset, defined as all participants who received at least one dose of the study intervention. To ensure appropriate patient selection for the composite endpoints, additional inclusion criteria were applied. For the SPFS endpoint, which required both PFS assessments and PRO scores throughout the study, patients were required to have available PFS data and at least one baseline and one post-baseline assessment on any PRO scale (e.g., pain, fatigue, mobility, drowsiness as measured by European Organization for Research and Treatment of Cancer (EORTC) Quality of Life Questionnaire-Core 30 (QLQ-C30) and/or EORTC Quality of Life Multiple Myeloma Questionnaire (MY20). Patients who lacked either a baseline or a post-baseline PRO score on all scales were excluded.

### Outcome measures

MM symptoms found to be relevant to patients and linked to disease progression that were identified in the SLR, and confirmed by the Delphi panel were subsequently identified in the MM-3 data collected through validated EORTCquestionnaires. Symptoms included pain (QLQ-C30), fatigue (QLQ-C30), poor mobility (QLQ-C30), and drowsiness (QLQ-MY20) [24, 25]. Descriptions and interpretations of these measures are displayed in Additional file 2. PRO questionnaires were administered during trial screening, on day (D) 1 of each 28-day cycle (C) and on D15 for C1-3, and on D1 only for C4 through Year 1. After 1 year, the questionnaires were administered every 3 cycles (C12D1, C15D1.) [24–27]. PRO scores (ranging from 0 to 100) were analyzed as observed values and as changes from baseline at each visit throughout MM-3 follow-up. High pain, fatigue, and drowsiness scores reflected worse quality of life, whereas low poor mobility scores were reflective of worse quality of life.

PFS was defined as the time from the date of first dose until confirmed progressive disease (PD) per IMWG criteria as assessed by Blinded Independent Central Review (BICR), or death due to any cause, whichever occurs first. SPFS was defined as clinical progression accompanied by a 10-point worsening of within-patient change from baseline in PRO scores for pain, fatigue, or poor mobility ( corresponding to the meaningful within-patient change [MWPC ]), within 28 days of the PFS event, or death, whichever occurs first [24, 28]. 

### Statistical methodology

The study analytical framework was designed to examine the relationship between longitudinal PRO symptom trajectories and time-to-event clinical outcomes, including disease progression or death, with the overarching goal of developing a comprehensive endpoint that integrates both PRO dynamics and survival data. Standard joint models were employed to quantify the relationship between changes in symptoms over time and the risk of progression or mortality, offering a more comprehensive and patient-centric understanding of how symptom worsening correlates with clinical deterioration [14–16]. 

Joint models were implemented separately for selected PRO symptom domains: QLQ-C30 pain, fatigue, poor mobility, and MY-20 drowsiness. Each joint model contained two sub-models, connected via a link function:

1. Survival sub-model: A Cox proportional hazards regression model to analyze PFS, defined as the time to first occurrence of disease progression or death [29].
2. Longitudinal sub-model: A linear mixed-effects model was applied to repeated PRO measurements, characterizing temporal trajectories of symptom severity while accounting for within-patient correlations and individual variability. PRO scores were entered as numerical values of change from baseline for each assessment (QLQ-C30 pain, fatigue, poor mobility, and MY-20 drowsiness) [30–32].

Covariates for both the Cox model and the linear mixed-effects model included age, sex, Eastern Cooperative Oncology Group (ECOG) performance status, disease stage, cytogenic risk, extramedullary disease, number of prior lines of therapy, penta-drug exposure, penta-drug refractory exposure, and time from diagnosis.

Several link functions were tested to quantify the relationship between the longitudinal model and survival model. The statistical framework for these models, the link functions and the criteria to select the best performing models is provided in Additional file 3 [33, 34].

Key outputs from the joint model include the regression coefficient $\:{\upeta\:}$, which measures the association between the PRO score and PFS. From $\:{\upeta\:}$, the percent increase in risk of progression or death corresponding to a 10-point change in PRO score (MWPC) was calculated.

Among the four symptoms of interest (pain, fatigue, poor mobility, and drowsiness), pain, fatigue and poor mobility had a statistically significant association with PFS and were included in the SPFS definition. Therefore, SPFS is defined as clinical progression accompanied by a 10-point worsening in PRO scale (pain, fatigue or poor mobility) from baseline, within 28 days of the PFS event, or death, whichever comes first. Patients without PD or death were right censored at the date of their last PRO assessment, while patients with PD but no symptom worsening from baseline within 28 days of PD were censored at PD + 28 days. SPFS was analyzed using the Kaplan–Meier method [35]. 

### Sensitivity analysis

To test the robustness of the SPFS composite endpoint definition, a sensitivity analysis was conducted where the time-window for PRO deterioration was extended. For this analysis, SPFS is defined as either clinical progression accompanied by a 10-point worsening in the PRO scale from baseline, occurring within 28 days before clinical progression or any time after clinical progression, or death.

## Results

### Identification of relevant MM symptoms

**Fig. 1:**
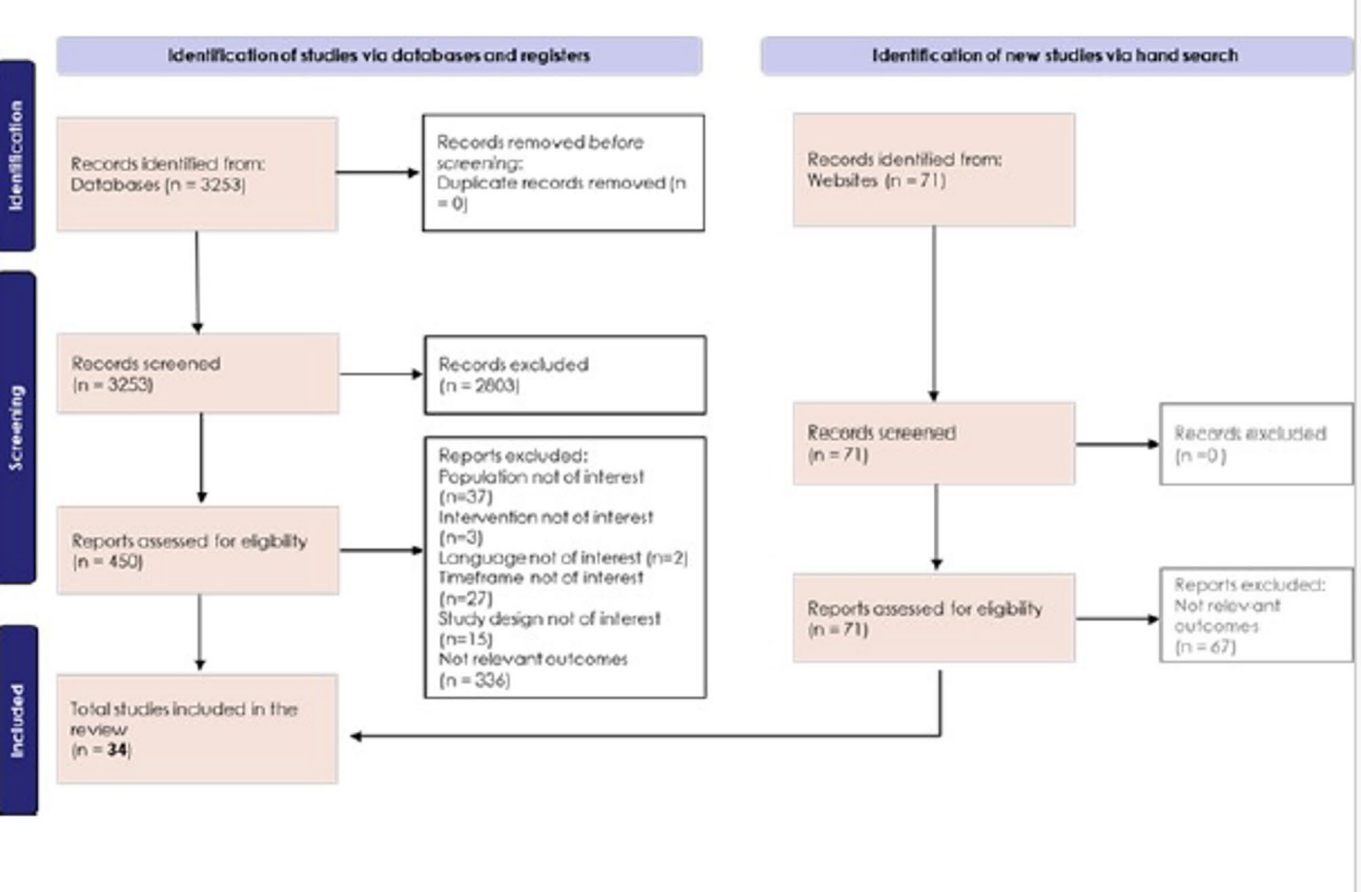
SLR screening process and publication results

The SLR yielded 3253 publications, resulting in 450 publications for full-text review, after title and abstract screening. Full-text review resulted in 416 studies being excluded due to outcomes (*n* = 336), population (*n* = 37), timeframe (*n* = 27), study design (*n* = 15), intervention (*n* = 3), or language (*n* = 2) not being of interest. This resulted in a final yield of 34 publications selected for inclusion (Fig. 1). Symptoms identified as being important to patients with MM and related to MM progression included: anxiety, appetite loss, constipation, depression, tingling in the hands and feet, peripheral neuropathy, poor mobility, fatigue, insomnia, mouth problems, nausea/vomiting, pain, diarrhea, difficulties remembering, drowsiness, and dyspnea/breathlessness.

The Delphi panel reached a consensus that pain and poor mobility notably affect patient quality of life (QoL). All panelists concurred that pain has a ‘very high’ impact on QoL, while they also agreed that poor mobility has either a ‘high’ or ‘very high’ impact. The panel additionally agreed that drowsiness, fatigue, pain, and poor mobility are associated with the progression of MM. There was 100% agreement for pain, 80% agreement for fatigue, and 70% agreement for poor mobility and drowsiness.

### MM-3 post-hoc analysis

For the 123 patients in Cohort A of the MM-3 trial, additional inclusion criteria were applied to ensure data availability for SPFS analysis, which relied on PFS assessments and PRO scores throughout the study. Patients were included if they:

1. Had available PFS data.
2. Completed at least one baseline and one post-baseline assessment on at least one PRO scale (e.g., pain, fatigue, mobility, or drowsiness measured by QLQ-C30 and/or MY20).

As a result, 114 patients met the criteria and were included in the final analysis (Table 2).

Mean (SD) age of participants was 67 (9) years, 54% of patients were male, and 36% of patients had an ECOG performance status of 0. Most patients were either disease stage I or II (79%) and 67% of patients were classified as having standard cytogenetic risk. Extramedullary disease (EMD) was present in 30% of patients. Finally, 66% of patients received five or fewer prior lines of therapy.

**Table 2:** MM-3 study population baseline characteristics

Variables	Category/statistic	Cohort A (*N* = 114)
Age	Mean (SD)	67 (9)
	Q1	63
	Median (Range)	68 (36, 9)
	Q3	73
Age, n (%)	[18–65]	39 (34.0%)
	[65, 75]	53 (46.0%)
	≥ 75	22 (19.0%)
Gender, n (%)	Male	62 (54.0%)
	Female	52 (46.0%)
Race, n (%)	White	65 (57.0%)
	Black or African American	9 (7.9%)
	Asian	16 (14.0%)
	American Indian or Alaska Native	0 (0.0%)
	Native Hawaiian or Other Pacific Islander	0 (0.0%)
	Multiracial	0 (0.0%)
	Not reported	23 (20.0%)
	Unknown	1 (0.9%)
Ethnicity, n (%)	Hispanic or Latino	6 (5.3%)
	Not Hispanic or Latino	82 (72.0%)
	Not reported	25 (22.0%)
	Unknown	1 (0.9%)
Geographic region, n (%)	North America	55 (48.0%)
	Europe	40 (35.0%)
	Asia	12 (11.0%)
	Other	7 (6.1%)
ECOG Performance Status, n (%)	0	41 (36.0%)
	1	67 (59.0%)
	2	6 (5.3%)
	3	0 (0.0%)
	Missing	0 (0.0%)
Disease Stage (R-ISS), n (%)	I	26 (23.0%)
	II	64 (56.0%)
	III	16 (14.0%)
	Unknown	8 (7.0%)
	Missing	0 (0.0%)
Cytogenetic Risk, n (%)	Standard Risk	76 (67.0%)
	High-Risk	29 (25.0%)
	Missing Data	9 (7.9%)
EMD by BICR, n (%)	Yes	34 (30.0%)
	Target EMD	32 (28.0%)
	Non-Target EMD only	2 (1.8%)
	Missing	0 (0.0%)
	No	80 (70.0%)
	Non-target Bone Lesions Only	54 (47.0%)
	No lesion	26 (23.0%)
	Missing	0 (0.0%)
Prior lines of therapy, n (%)	≤ 5	75 (66.0%)
	*>* 5	39 (34.0%)

*BICR* blinded independent central review, *EMD* extramedullary disease, *ECOG* Eastern Cooperative Oncology Group, *n* number of participants, *Q1* first quartile, *Q3* third quartile, *R-ISS* Revised International Staging System, *SD* standard deviation

### Standard joint model

For the joint models, for all scales except poor mobility, models with the lowest DIC/WAIC values successfully converged after 3,500 iterations. However, for poor mobility, the model with the lowest DIC/WAIC did not converge adequately and alternative models with slightly higher DIC/WAIC values were tested. For all scales, optimal models used a cumulative effect as the link function between the two sub-models.

Statistically significant associations were observed between PRO trajectories and PFS in the QLQ-C30 domains for pain, fatigue, and poor mobility (Table 3), as indicated by credible intervals that do not include 0. A 10-point worsening on the 100-point pain scale for pain from baseline corresponded to a 22% increase in risk of progression or death. Similarly, a 10-point worsening in fatigue and poor mobility scores was associated with a 20% and 31% increase in the risk of progression or death, respectively. Changes in drowsiness PRO scores did not demonstrate a statistically significant impact on the risk of disease progression or death. Regression coefficients and corresponding credible intervals are described in Additional file 4 and Additional file 5.

**Table 3:** Impact of PRO score trajectories on PFS

PRO domain	Coefficient of association between PRO score and PFS (95% CrI)	% increase in the risk of PD or death corresponding to a 10-point worsening in PRO score * (posterior tail probability**)
QLQ-C30, pain	0.022 (0.008 to 0.036)	22% (0.003)
QLQ-C30, fatigue	0.018 (0.002 to 0.034)	20% (0.022)
QLQ-C30, poor mobility	−0.027 (- 0.042 to - 0.013)	31% (0.000)
MY20, drowsiness	0.011 (- 0.020 to 0.041)	11% (0.490)

*CrI* credible interval, *MY20*, Multiple Myeloma Questionnaire 20, *PD* progressive disease, *PFS*, progression-free survival, *PRO* patient-reported outcome, *QLQ-C30* (Quality of Life Questionnaire-Core 30

*% risk calculated by: exp (0.022*10) = 1.22

**The probability of the effect being in the direction opposite to the estimate

### Symptomatic progression-free survival

**Fig. 2:**
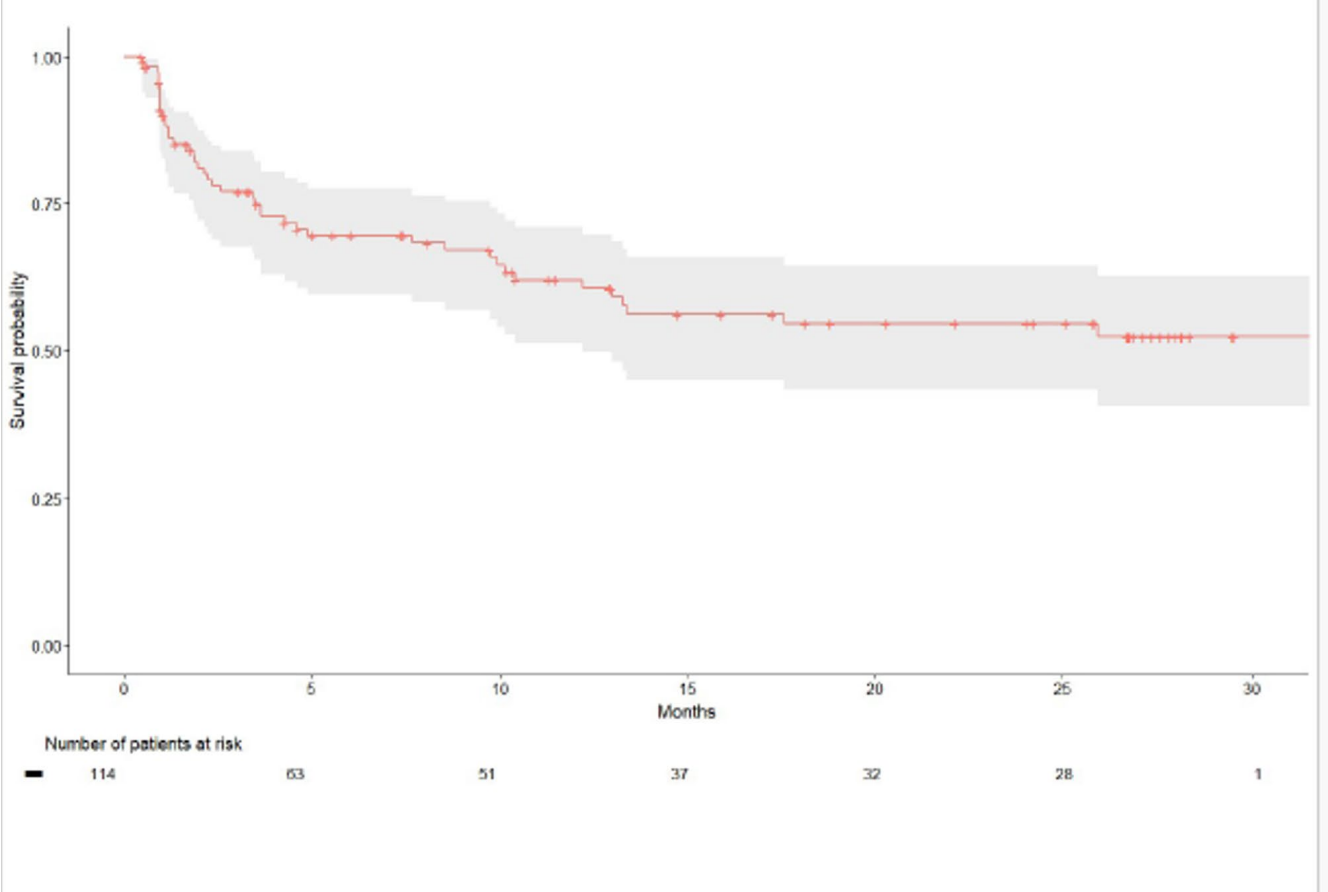
Symptomatic progression-free survival (SPFS). Shaded bands indicate 95% confidence intervals

The median (IQR) follow-up time was 7.54 months (1.66, 24.17). In total, 43 patients (38%) experienced symptomatic progression, defined by PD and a deterioration of at least 10 points from baseline in either EORTC QLQ-C30 pain, fatigue, or poor mobility within 28 days of confirmed PD, or death, whichever occurred first (Table 4; Fig. 2). In contrast, only 8 patients (7%) experienced progression without a concurrent PRO deterioration. Median SPFS was not reached (Table 4).

**Table 4:** SPFS event rates

SPFS	Category	Cohort A (*N* = 114)
Events	*SPFS event*	43 (38%)
	PD and deterioration in pain, fatigue,0 or poor mobility within 28 days of PD	24 (21%)
	Death	19 (17%)
Censoring	*Censored*	71 (62%)
	PD and no deterioration 28 days around PD	8 (7.0%)
	PD or death after missing or inadequate post-baseline assessments	3 (2.6%)
	Ongoing without PD or death	39 (34%)
	Start of new anticancer therapy	16 (14%)
	Withdrawal of consent	5 (4.4%)

*PD* progressive disease, *SPFS* symptomatic progression-free survival

### Sensitivity analysis

Results of the sensitivity analysis were similar to the base case (Table 5). In total, 44 patients (39%) experienced symptomatic progression, defined by PD and a deterioration of at least 10 points from baseline in either EORTC QLQ-C30 pain, fatigue, or poor mobility 28 days prior to confirmed PD, or death, or any time after (Table 4). Similarly to the base case, only 7 patients (versus 8 patients in the base case) experienced progression without a concurrent PRO deterioration.

**Table 5:** Sensitivity analysis 1: SPFS results

SPFS	Category	Cohort A (*N* = 114)
Events	*SPFS event*	44 (39%)
	PD and deterioration 28 days around PD	25 (22%)
	Death	19 (17%)
Censoring	*Censored*	70 (61%)
	PD and no deterioration 28 days around PD	7 (6.1%)
	PD or death after missing or inadequate post-baseline assessments	3 (2.6%)
	Ongoing without PD or death	39 (34%)
	Start of new anticancer therapy	16 (14%)
	Withdrawal of consent	5 (4.4%)

SPFS is defined as confirmed PD or death per IMWG criteria as assessed by BICR and a degradation > = 10 points (MWPC) in > = 1 of the following PRO domains < = 28 days prior to PD or any time after: EORTC QLQ-C30– Pain, EORTC QLQ-C30– Fatigue, EORTC QLQ-C30– poor mobility

*PD* Progressive Disease, *SPFS* Symptomatic progression-free survival

## Discussion

In this study, we adopted a systematic approach to develop a novel endpoint that integrates both clinical disease progression and patient symptom trajectories. This approach leveraged validated methodologies, including a comprehensive SLR to identify key symptoms relevant to patients, and a Delphi panel to gain expert consensus on symptoms likely linked to disease progression. The analysis of MM-3 trial data provided valuable insights into the relationship between these symptoms and PFS, using a joint model to quantify this relationship. These rigorous techniques ensure a robust and clinically meaningful endpoint that captures the multidimensional nature of disease progression and patient experience, allowing for the construction and evaluation of the SPFS composite endpoint.

Results from the joint model suggest that there is a relationship between PRO scores (for fatigue, pain, and poor mobility) and progression or death. Inclusion of the three statistically significant symptoms (pain, fatigue, and poor mobility) in the composite endpoint with PFS resulted in a new patient-relevant endpoint of SPFS (i.e., disease progression and a 10-point worsening from baseline in at least one of the symptoms within 28 days of progression, or death, whichever comes first). After a median follow-up of 7.5 months, median SPFS was not reached.

While this work is a unique standalone study, our SPFS endpoint can be leveraged in future studies to provide additional value in understanding the efficacy of treatment options in MM. The construction of a novel endpoint, SPFS, represents an important step toward patient-centered clinical evaluation in MM. By incorporating significant symptom deterioration alongside traditional clinical measures, this endpoint offers a more holistic view of disease progression that aligns closely with patient experiences. This may enhance the clinical relevance of progression-based endpoints by better capturing the real-world impact of treatment and guide treatment decisions, ultimately reflecting both clinical and symptomatic outcomes.

The analysis showed that symptomatic progression closely mirrored the clinical endpoints of PFS, suggesting that clinical progression may already encompass patient-relevant outcomes. Our results suggest that for the majority of patients with progression, PRO deterioration was accompanied by a meaningful decline in symptoms as reported by patients. However, the smaller subset (7%) who progressed without PRO deterioration may indicate cases where clinical progression did not immediately translate into noticeable symptom worsening, potentially highlighting heterogeneity in patient experiences or differences in the sensitivity of clinical versus PRO measures.

This study demonstrated several strengths, including its systematic approach to endpoint development, which integrated clinical disease progression and patient-reported symptom trajectories. The comprehensive literature review and Delphi panel consensus contributed validity to the selection of key symptoms linked to progression. Capturing the nuanced impact of disease progression on QoL through SPFS, could be especially valuable for HTAs through demonstration of the multifaceted (i.e., clinical and patient focussed) impact of a treatment. This endpoint could additionally help address previous skepticism regarding the patient relevance of PFS [36], thus strengthening the overall evaluation of treatment efficacy in MM. The sensitivity analysis supported the robustness of the findings, showing consistent results with the base case.

The study had some limitations, including its relatively small sample size and the low number of patients at risk for disease progression beyond 20 months, which may limit the generalizability of the findings compared to larger comparative studies, such as those by Knop et al. 2024 [12]. The low number of patients at risk for disease progression beyond this time point, coupled with a median follow-up of 7.5 months (range, 0.46 to 34.99 months), meant that the median SPFS was not reached. Consequently, further research with a larger sample size of patients with MM is needed to confirm these findings. Additionally, we excluded patients with early mortality since they often lack sufficient post-baseline PRO data to assess within-patient change and this may have introduced selection bias.

An important limitation relates to the evolving therapeutic landscape in MM. The high efficacy of new treatments has driven the adoption of highly sensitive response measures, such as MRD, for disease monitoring and decision-making. However, MRD is currently recognized primarily in the context of conditional regulatory approvals and does not capture the patient experience [37]. Therefore, symptom data correlated with clinical progression remain essential to fully reflect treatment impact on QoL. Finally, although the Delphi panel incorporated the perspectives of global MM experts, patient input regarding the relevance of symptoms was not considered. Further studies involving patient feedback in a replicated Delphi panel is warranted to assess alignment with the expert consensus on symptom relevance.

## Conclusions

This study underscores the importance of a patient-centric approach in the evaluation of MM treatments. By integrating PROs, this work aligns trial outcomes more closely with patient experiences, potentially improving the relevance of clinical trial endpoints for patients, clinicians, and HTA bodies. While this was a single-arm, non-randomized study, further research could explore the integration of these novel patient-centric endpoints into comparative clinical trial designs. Such efforts could further improve the relevance of clinical outcomes to patient QoL and provide deeper insights into the real-world impact of treatment options, ultimately aiding in the development of more effective, patient-centered therapeutic strategies.

## Supplementary Information

Below is the link to the electronic supplementary material.

Supplementary Material 1

Supplementary Material 2

Supplementary Material 3

Supplementary Material 4

Supplementary Material 5

Supplementary Material 6

## Data Availability

Upon request, and subject to review, Pfizer will provide the data that support the findings of this study. Subject to certain criteria, conditions and exceptions, Pfizer may also provide access to the related individual de-identified participant data. See https://www.pfizer.com/science/clinical-trials/trial-data-and-results for more information.

## Abbreviations

BCMA: B-cell maturation antigen
BICR: Blinded independent central review
C: Cycle
D: Day
DIC: Deviance information criterion
ECOG: Eastern cooperative oncology group
EMD: Extramedullary disease
EORTC: European organization for research and treatment of cancer
G-BA: Gemeinsamer bundesausschuss
HTA: Health technology assessment
IMiD: Immunomodulatory drug
IMWG: International myeloma working group
IQR: Interquartile range
mAB: Monoclonal antibody
MM: Multiple myeloma
MM-3: MagnetisMM-3
MWPC: Meaningful within-patient change
NICE: National Institute for health and care excellence
NR: Not reached
Q1: First quartile
Q3: Third quartile
OS: Overall survival
PD: Progressive disease
PFS: Progression-free survival
PI: Proteasome inhibitor
PICOS: Population, interventions, comparator, outcomes and study
PRO: Patient-reported outcome
QLQ-C30: Quality of Life Questionnaire-Core 30
QLQ-MY20: Quality of life multiple myeloma questionnaire
R-ISS: Revised International staging system
RRMM: Relapsed, refractory multiple myeloma
RCT: Randomised clinical trial
SD: Standard deviation
SLR: Systematic literature review
SPFS: Symptomatic progression free survival
WAIC: Watanabe–Akaike Information Criterion
