# Supplementary material for: Symptomatic progression-free survival as an emerging patient-centered endpoint in multiple myeloma: a secondary analysis of MagnetsiMM-3 trial data
Source: BMC Cancer. 2025 Aug 8;25:1288. doi: 10.1186/s12885-025-14724-6 (PMC12333233; doi:10.1186/s12885-025-14724-6)
Supplement: Supplementary file 1 — Supplementary Material 1 [file 12885_2025_14724_MOESM1_ESM.docx]

**SLR search strategy**

| **No** | **Query** | **Results** |
| --- | --- | --- |
| **#1** | 'multiple myeloma'/exp OR 'multiple myeloma':ti,ab | 107863 |
| **#2** | 'symptom'/exp OR 'symptom*':ti,ab | 2128198 |
| **#3** | 'signs and symptoms'/de | 13266 |
| **#4** | ('quality of life' OR qol) NEAR/3 (symptom* OR event* OR sign* OR perspective* OR experience* OR prognosis OR prognostic OR progression OR progress) | 71731 |
| **#5** | symptom* NEAR/3 (prognosis OR prognostic OR progression OR progress OR experience* OR perspective* OR burden OR impact OR risk OR prediction) | 89474 |
| **#6** | symptom* AND progression | 92834 |
| **#7** | 'pain'/de OR 'fatigue'/de OR 'tiredness'/de OR 'depressive disorders' OR 'drowsiness'/de OR 'weakness'/de OR 'lack of energy'/de OR 'appetite'/de OR 'anorexia'/de OR 'dyspnea'/de OR 'shortness of breath'/de OR 'depressed mood'/de OR 'sadness'/de OR 'hopelessness'/de OR 'anxiety'/de OR 'nervousness'/de OR 'worrying'/de OR 'nausea'/de OR 'vomiting'/de OR 'sleeplessness'/de OR 'confusion'/de OR 'delirium'/de OR 'constipation'/de OR 'obstipation'/de OR 'diarrhea'/de OR 'dry mouth'/de OR 'sore mouth'/de OR 'edema'/de OR 'oedema'/de OR 'swelling'/de OR 'restlessness'/de OR 'loss of mobility' OR 'dysfunctional' OR 'cough'/de OR 'discomfort'/de OR 'distress'/de | 1966810 |
| **#8** | #2 OR #3 OR #4 OR #5 OR #6 OR #7 | 3661286 |
| **#9** | 'randomized controlled trial'/de OR 'controlled clinical trial'/de OR 'randomization'/de OR 'random*':ab,ti OR 'placebo*':ab,ti OR ((open NEXT/1 label):ti,ab) OR (((double OR single OR doubly OR singly OR treble OR triple) NEXT/1 (blind OR blinded OR blindly)):ti,ab) OR 'single blind procedure'/de OR 'double blind procedure'/de OR ((parallel NEXT/1 group*):ti,ab) OR (((assign* OR match OR matched OR allocation) NEAR/6 (alternate OR group OR groups OR intervention OR interventions OR patient OR patients OR subject OR subjects OR participant OR participants)):ti,ab) OR assigned:ti,ab,tt OR allocated:ti,ab OR ((controlled NEAR/8 (study OR design OR trial)):ti,ab) OR 'multicenter study'/de OR trial:ti | 3141641 |
| **#10** | 'clinical study'/de OR 'observational study'/de OR 'case control study'/de OR 'retrospective study'/de OR 'cohort analysis'/de OR 'prospective study'/de OR 'cross-sectional study'/de OR 'longitudinal study'/de | 3684344 |
| **#11** | (cohort OR 'case control' OR 'follow up' OR observational OR prospective OR retrospective OR 'cross sectional' OR 'natural history') NEAR/1 (study OR studies) | 3517056 |
| **#12** | 'cohort analy*':ti,ab OR 'case control':ti,ab OR 'cross sectional':ti,ab | 860620 |
| **#13** | 'natural history':ti,ab OR ((natural OR disease) NEAR/3 (history OR course)) OR ('natural history' NEAR/3 ('disease' OR 'course' OR 'progress*')) | 691193 |
| **#14** | 'qualitative research'/de | 112696 |
| **#15** | 'focus group*' OR 'interview*' OR 'qualitative' | 931711 |
| **#16** | #1 AND #8 AND #9 | 3647 |
| **#17** | #1 AND #8 AND (#10 OR #11 OR #12) | 3692 |
| **#18** | #1 AND #8 AND #13 | 1729 |
| **#19** | #1 AND (#14 OR #15) | 978 |
| **#20** | #16 OR #17 OR #18 OR #19 | 8237 |
| **#21** | (#16 OR #17 OR #18 OR #19) AND [2013-2023]/py | 5762 |
| **#22** | 'animal experiment'/de NOT ('human experiment'/de OR 'human'/de) | 2526561 |
| **#23** | 'case study'/exp OR 'case study' OR 'case report':ti,ab OR 'letter'/exp OR 'letter' OR 'editorial':it OR 'letter':it OR 'note':it | 3743524 |
| **#24** | #21 NOT (#22 OR #23) | 5454 |
| **#25** | #24 AND ([article]/lim OR [article in press]/lim) AND [english]/lim | 2289 |
