# Supplementary material for: Symptomatic progression-free survival as an emerging patient-centered endpoint in multiple myeloma: a secondary analysis of MagnetsiMM-3 trial data
Source: BMC Cancer. 2025 Aug 8;25:1288. doi: 10.1186/s12885-025-14724-6 (PMC12333233; doi:10.1186/s12885-025-14724-6)
Supplement: Supplementary file 2 — Supplementary Material 2 [file 12885_2025_14724_MOESM2_ESM.pdf]

Statistical framework for joint models

The statistical framework for the standard joint model is described below.[13–15]

Let us denote by $i, i = 1, \dots N$, individuals in a set of data, T_i the observed event time, C_i the censoring status, T_i^* the true event time of interest and $\delta_i = 1_{\{T_i^* \leq C_i\}}$ the indicator of event. Let us also define $Y_i = (Y_i(t_{i1}), \dots, Y_i(t_{in_i}))$a n_i-vector corresponding to a specific PRO score. We denote by $X_L(t)$ a (n, p) time dependent fixed-effect covariate matrix for the longitudinal model, $Z(t)$ a (n, q) time dependent random-effect matrix, and X_S a (n, r) matrix in the survival model. In the joint model (JM) we consider the following sub-models:

Linear mixed-effects model sub-model:

$$\begin{aligned} Y_i(t_{ij}) &= Y_i^*(t_{ij}) + \epsilon_{ij} \\ &= X_{Li}(t_{ij})^T \beta + Z_i(t_{ij})^T b_i + \epsilon_i(t_{ij}), \end{aligned}$$

with:

- $Y_i^*(t_{ij})$ the "true" unobserved trajectory of PRO for $j = 1, \dots n_i$. In the linear mixed-effects model, we assume that $Y_i(t_{ij})$ is a noisy measure of the "true" unobserved trajectory.
    - β the p-vector associated with fixed effects and b_i the q-vector associated with the random effect such that $b_i \sim N(0, B)$ and B an unstructured variance-covariance matrix (chosen as the most flexible matrix to model the relationship among random effects in mixed effect model).
    - $\epsilon_i = \left(\epsilon(t_{i1}), \dots, \epsilon(t_{in_i})\right)^T$ the vector of residuals corresponding to the error of measurement of the PRO such that $\epsilon_i \sim N(0, \sigma^2 I_{n_i})$ where $\sigma^2 I_{n_i}$ is the variance-covariance matrix.
    - $X_{Li}(t_{ij})^T \beta$ corresponds to the mean trajectory of the PRO overtime while, $Z_i(t_{ij})^T b_i$ represents the individual deviation relative to the mean trajectory. $X_{Li}(t_{ij})$ and $Z_i(t_{ij})$ are respectively p and q vector of time dependent covariates of fixed and random effect of individual i at time t.

Key assumptions of this model include:

- ϵ_i and b_i are independent.
- $Y_i \sim N(X_{Li}\beta + Z_i b_i, \sigma^2 I_{n_i})$.
    - Cox proportional sub-model, under the assumption of proportional hazards:

$$\lambda_i(t|X_{Si}, b_i) = \lambda_0(t) \ \exp\left(X_{Si}^T \gamma + h(b_i, t)^T \eta\right),$$

with:

- $\lambda_0(t)$ corresponding to the baseline hazard function, no parametric assumption is made on its form.

- γ the r-vector of coefficient quantifying the association between variables of interest in X_S (the same variables included in fixed effect X_{Li} variable) and the survival outcome.

- $h(.)$ represents a multivariate function linking the survival outcome and the PRO. This function is a key component of the joint model as it reflects the type of relationship assumed between both components.[14,38] This function could specified as follows:

  - The current "true" unobserved (latent) trajectory of the PRO: $h(b_i, t) = Y_i^*(t)$.
  - The slope of the current trajectory of the PRO score: $h(b_i, t) = \frac{\partial Y_i^*(t)}{\partial t}$
  - Both the current value of the PRO score and the trajectory of the slope $h(b_i, t) = Y_i^*(t) + \frac{\partial Y_i^*(t)}{\partial t}$.
  - Both the current value of the PRO score and the trajectory of the next slope $h(b_i, t) = Y_i^*(t) + \frac{\partial Y_i^*(t+1)}{\partial t+1}$.
  - Both the current value of the PRO score and the trajectory of the previous slope $h(b_i, t) = Y_i^*(t) + \frac{\partial Y_i^*(t-1)}{\partial t-1}$.
  - The cumulative effect of the PRO score trajectory up to time t: $h(b_i, t) = \int_0^t \frac{Y_i^*(s)ds}{t}$
- η corresponds to the vector of coefficients quantifying the association between the PRO and the survival outcome associated to the multivariate function h(.).

<u>Standard Joint model:</u>

The distribution of the joint model is defined as follows[39,40] :

$$p(y_i, T_i, \delta_i) = \int_b f_Y(Y_i|X_{Li}, b_i; \theta)\{f_T(T_i|X_{Si}, b_i; \theta)\ f_b(b_i; \theta)\}db_i$$

$$= \int_b f_Y(Y_i|X_{Li}, b_i; \theta)\{\lambda_i(T_i|X_{Si}, b_i; \theta)^{\delta_i} S_i(T_i|X_{Si}, b_i; \theta) f_b(b_i; \theta)\}db_i$$

with:

- θ the set of all parameters of the joint model.
- f_b is the multivariate Gaussian density function of b_i previously defined.
- f_Y is respectively the multivariate Gaussian density function of Y.
- b_i is the random effect q-vector defined previously.
- $\lambda_i(T_i|X_{Si}, b_i; \theta)$ is the hazard function defined previously.
- $S_i(T_i|X_{Si}, b_i; \theta) = e^{-\int_0^{T_i} (\lambda_i(t|X_{S_i}, b_i; \theta)}$ represents the derived survival functions.

  The key assumptions of the joint model are the following:[41]
- Both survival and longitudinal components are conditionally independent given the random effects defined in the LLM.
- The longitudinal outcome is independent of the time-to-event outcome.
