# Supplementary material for: Symptomatic progression-free survival as an emerging patient-centered endpoint in multiple myeloma: a secondary analysis of MagnetsiMM-3 trial data
Source: BMC Cancer. 2025 Aug 8;25:1288. doi: 10.1186/s12885-025-14724-6 (PMC12333233; doi:10.1186/s12885-025-14724-6)
Supplement: Supplementary file 3 — Supplementary Material 3 [file 12885_2025_14724_MOESM3_ESM.pdf]

PRO description

Symptoms of interest	Symptom specific PRO items	Cluster of PROs	Score interpretation	MWPC
EORTC QLQ-C30				
Pain	9. Have you had pain?	Symptom scale	Linear transformation was used to standardize the raw score. Scores range from 0 to 100.<br>For ***Symptom scale:*** a higher score represents a **worse** level of symptom	A Minimal Clinically Important Difference of **5 to 10 points**, as defined by several studies[27,36], is generally recommended for interpreting group differences and changes in the EORTC QLQ-C30 scale scores as clinically meaningful.
	19. Did pain interfere with your daily activities?			
Fatigue	10. Did you need to rest?			
	12. Have you felt weak?			
	18. Were you tired?			
Poor mobility	1. Do you have any trouble doing strenuous activities, like carrying a heavy shopping bag or a suitcase?	Functional scale	Linear transformation used to standardize the raw score. That score ranges from 0 to 100.<br>For ***Functional scale:*** a higher score represents a **better** level of functioning	
	2. Do you have any trouble taking a long walk?			
	3. Do you have any trouble taking a short walk outside of the house?			
	4. Do you need to stay in bed or a chair during the day?			
	5. Do you need help with eating, dressing, washing yourself or using the toilet?			
EORTC MY20				
Drowsiness	37. Did you feel drowsy?	Treatment side-effects	Linear transformation used to standardize the row score. That score ranges from 0 to 100.<br>For ***Drowsiness***: a higher score represents a **worse** level of drowsiness	Recommended Minimal Clinically Important Difference for Side Effects of Treatment is 10 points[37]

Abbreviations: EORTC: European organization for research and treatment of cancer; MY20: Multiple myeloma 20, PRO: patient-reported outcome; QLQ-C30: Quality of life questionnaire-core 30
