# Supplementary material for: Symptomatic progression-free survival as an emerging patient-centered endpoint in multiple myeloma: a secondary analysis of MagnetsiMM-3 trial data
Source: BMC Cancer. 2025 Aug 8;25:1288. doi: 10.1186/s12885-025-14724-6 (PMC12333233; doi:10.1186/s12885-025-14724-6)
Supplement: Supplementary file 4 — Supplementary Material 4 [file 12885_2025_14724_MOESM4_ESM.pdf]

Performance measures of the selected models

PRO domain	DIC [1]	WAIC [2]	LPML [3]
QLQ-C30, pain	11071.39	11614.44	-6589.19
QLQ-C30, fatigue	10713.76	11154.65	-6329.63
QLQ-C30, poor mobility	10538.37	12477.56	-7679.60
MY20, drowsiness	9283.22	10186.28	-6249.05

Abbreviations: AIC: Akaike information criterion; DIC: deviance information criterion; LPML: logarithm of the pseudo marginal likelihood; MY20: Multiple Myeloma Questionnaire 20; PD: progressive disease; PRS: progression-free survival; PRO: patient-reported outcome; QLQ-C30: (Quality of Life Questionnaire-Core 30; WAIC: widely acceptable information criterion

[1]: DIC generalizes upon AIC and is typically used in Bayesian framework and hierarchical models.; ***[2]:*** WAIC improves upon the DIC by accounting for model uncertainty and out-of-sample predictive performance in the Bayesian framework), ***[3]*** LPML is a fully Bayesian criterion based on leave-one-out cross-validation, measuring predictive accuracy to select the best model

DIC and WAIC values and higher LPML values indicate better model performance.
