# Supplementary material for: Symptomatic progression-free survival as an emerging patient-centered endpoint in multiple myeloma: a secondary analysis of MagnetsiMM-3 trial data
Source: BMC Cancer. 2025 Aug 8;25:1288. doi: 10.1186/s12885-025-14724-6 (PMC12333233; doi:10.1186/s12885-025-14724-6)
Supplement: Supplementary file 5 — Supplementary Material 5 [file 12885_2025_14724_MOESM5_ESM.pdf]

Joint model: Output of the Cox regression

		Regression coefficients [95% CrI]: Cox regression			
		QLQ-C30 Pain (Cumulative effect)	QLQ-C30 Fatigue (Cumulative effect)	QLQ-C30 Poor mobility (Cumulative effect)	MY-20 Drowsiness (Cumulative effect)
	Age	0.015 [-0.029,0.058]	0.008 [0.035,0.05]	0.004 [-0.039, 0.045]	0.013 [-0.032, 0.061]
**Sex (Ref: Female)**	Male	0.38 [-0.241,0.994]	0.264 [-0.37,0.881]	0.388 [-0.228, 0.998]	0.382 [-0.222, 0.979]
**ECOG (ref: 0)**	ECOG 1	-0.331 [-0.949,0.318]	-0.262 [-0.872,0.391]	-0.465 [-1.106, 0.165]	-0.212 [-0.848,0.437]
	ECOG2	-0.497 [-2.499,1.153]	-0.359 [-2.327, 1.345]	-0.595 [-2.588, 1.095]	-0.352 [-2.371, 1.349]
**Disease stage (ref: I)**	II	0.385 [-0.418,1.213]	0.244 [-0.557,1.144]	0.236 [-0.528, 1.062]	0.34 [-0.507,1.195]
	III	0.772 [-0.353,1.868]	0.785 [-0.383, 1.937]	0.869 [-0.276, 2.049]	0.835 [-0.254,1.972]
	Unknown	0.522 [-0.912,1.832]	0.336 [-1.158, 1.708]	0.445 [-1.031, 1.798]	-0.005 [-1.617, 1.44]
**Cytogenic risk (ref: High risk)**	Missing Data	-0.599 [-1.81,0.472]	-0.396 [-1.565, 0.709]	-0.442 [-1.633, 0.65]	-0.263 [-1.609, 0.844]
	Standard Risk	-0.368 [-1.027,0.324]	-0.219 [-0.909, 0.503]	-0.292 [-0.971, 0.437]	-0.198 [-0.882, 0.514]
**Extramedullary Disease (ref: No)**	Yes	0.581 [-0.12,1.249]	0.481 [-0.213, 1.138]	0.426 [-0.304, 1.105]	0.515 [-0.214, 1.219]
**Number of prior line (ref: ≤5)**	> 5	-0.089 [-0.84,0.649]	0.004 [-0.7, 0.705]	-0.016 [-0.75, 0.722]	-0.27 [-1.066, 0.494]
**Penta-drug exposed (ref: No)**	Yes	-0.34 [-1.227,0.55]	-0.333 [-1.181, 0.528]	-0.362 [-1.145, 0.48]	-0.164 [-1.04, 0.743]
**Penta-drug refractory (ref. No)**	Yes	0.716 [-0.077,1.493]	0.701 [-0.054, 1.505]	0.583 [-0.146, 1.332]	0.692 [-0.051, 1.458]
	Time from diagnosis	0.002 [-0.007,0.011]	0.003 [-0.005, 0.011]	0.003 [-0.006, 0.011]	0.004 [-0.005,0.013]
	Association (η) between PRO and PFS	0.022 [0.008,0.036]	0.018 [0.002, 0.034]	-0.027 [-0.042, -0.013]	0.011 [-0.02, 0.041]

Abbreviations: CrI: Credible interval, ECOG: Eastern Cooperative Oncology Group, MY-20: Multiple Myeloma, QLQ-C30: Quality of Life Questionnaire-Core 30.

Note: The association between PRO and PFS row indicates the change in risk of PFS associated with a 1-point worsening in score of the corresponding PRO (e.g., a 1-point worsening in pain score was associated with a 2% increase in risk of disease progression or death, and therefore a 10-point worsening corresponds to a 22% increase in risk of progression or death).

An increase in PRO score for QLQ-C30 pain, QLQ-C30 fatigue, and MY-20 drowsiness scores indicates worsening, while a decrease indicates improvement; an increase in PRO scores for QLQ-C30 poor mobility indicates improvement, while a decrease indicates worsening.

For continuous predictors (e.g., Age, Time from diagnosis): Coefficients represent the estimated log hazard ratio for progression/death per one-unit increase in the predictor (positive = higher risk, negative = lower risk).

For categorical predictors (e.g., Sex, ECOG): Coefficients reflect the log hazard ratio difference compared to the reference group (e.g., Male vs. Female, ECOG 1 vs. ECOG 0).

Association (η): A positive η indicates that higher cumulative PRO scores correlate with increased risk of progression/death; a negative η suggests higher scores correlate with reduced risk of progression/death.
