# Supplementary material for: Symptomatic progression-free survival as an emerging patient-centered endpoint in multiple myeloma: a secondary analysis of MagnetsiMM-3 trial data
Source: BMC Cancer. 2025 Aug 8;25:1288. doi: 10.1186/s12885-025-14724-6 (PMC12333233; doi:10.1186/s12885-025-14724-6)
Supplement: Supplementary file 6 — Supplementary Material 6 [file 12885_2025_14724_MOESM6_ESM.pdf]

Joint model: Output of the linear mixed-effects model

		Regression coefficients [95% CrI]: linear mixed-effects model			
		QLQ-C30 Pain (Cumulative effect)	QLQ-C30 Fatigue (Cumulative effect)	QLQ-C30 Poor mobility (Cumulative effect)	MY-20 Drowsiness (Cumulative effect)
	Intercept	35.581 [6.432,64.973]	29.437 [4.661, 54.627]	92.052 [66.539, 117.491]	22.721 [6.38, 39.273]
	Month	0.012 [-0.224,0.231]	-0.355 [-0.587, -0.117]	0.124 [-0.135, 0.382]	-0.141 [-0.233, -0.046]
	Age	-0.037 [-0.459,0.389]	0.15 [-0.212, 0.511]	-0.276 [-0.644, 0.093]	-0.058 [-0.293, 0.175]
**Sex (Ref: Female)**	Male	-0.188 [-8.499,7.961]	6.376 [-0.462, 13.252]	1.181 [-5.902, 8.389]	2.138 [-2.092, 6.417]
**ECOG (ref: 0)**	ECOG 1	2.579 [-6.393,11.398]	4.415 [-3.062, 11.579]	-6.855 [-14.405, 0.651]	3.597 [-0.964, 8.155]
	ECOG2	0.978 [-18.523,20.147]	-4.858 [-21.252, 11.517]	-7.214 [-23.973, 9.43]	0.423 [-10.779, 11.712]
**Disease stage (ref: I)**	II	-3.766 [-14.252,6.4]	3.501 [-4.961, 11.83]	-0.732 [-9.57, 8.021]	0.485 [-4.806, 5.758]
	III	-5.535 [-19.93,8.629]	3.735 [-8.242, 15.655]	0.407 [-11.908, 12.776]	-0.274 [-7.855, 7.188]
	Unknown	-8.923 [-26.922,8.683]	2.588 [-12.638, 17.491]	5.193 [-10.11, 20.388]	2.972 [-6.656, 12.308]
**Cytogenic risk (ref: High risk)**	Missing Data	13.641 [-3.438,30.968]	13.399 [-0.701, 27.909]	-10.82 [-25.338, 3.656]	8.656 [-0.247, 17.791]
	Standard Risk	0.306 [-9.311,10.129]	-4.764 [-12.863, 3.473]	3.392 [-4.907, 11.752]	-1.083 [-6.136, 3.895]
**Extramedullary Disease (ref: No)**	Yes	1.73 [-7.607,11.144]	1.98 [-6.256, 10.351]	-6.144 [-14.865, 2.332]	-2.489 [-7.296, 2.433]
**Number of prior line (ref: ≤5)**	> 5	5.618 [-4.514,15.764]	-0.366 [-8.75, 7.769]	-2.304 [-11.005, 6.296]	0.923 [-4.399, 6.273]
**Penta-drug exposed (ref: No)**	Yes	2.642 [-7.861,12.999]	2.704 [-5.975, 11.218]	-1.396 [-10.372, 7.447]	4.103 [-1.416, 9.628]
**Penta-drug refractory (ref. No)**	Yes	-2.632 [-12.376,7.277]	-1.676 [-9.637, 6.504]	-1.03 [-9.428, 7.231]	-2.613 [-7.64, 2.451]
	Time from diagnosis	-0.044 [-0.147,0.06]	-0.05 [-0.134, 0.036]	0.035 [-0.052, 0.127]	-0.049 [-0.103, 0.005]

Abbreviations: CrI: Credible interval, ECOG: Eastern Cooperative Oncology Group, MY-20: Multiple Myeloma, QLQ-C30: Quality of Life Questionnaire-Core 30.
Note: An increase in PRO score for QLQ-C30 pain, QLQ-C30 fatigue, and MY-20 drowsiness scores indicates worsening, while a decrease indicates improvement. An increase in PRO scores for QLQ-C30 poor mobility indicates improvement, while a decrease indicates worsening.
For continuous predictors (e.g., Month, Age): Coefficients represent the estimated average change in the PRO score per one-unit increase in the predictor (positive = higher score, negative = lower score).
For categorical predictors (e.g., Sex, ECOG): Coefficients reflect the estimated difference in the outcome score between the category and its reference group (e.g., Male vs. Female, ECOG 1 vs. ECOG 0).
